# Supplementary material for: A nuclease-mimetic platinum nanozyme induces concurrent DNA platination and oxidative cleavage to overcome cancer drug resistance
Source: Nat Commun. 2022 Nov 30;13:7361. doi: 10.1038/s41467-022-35022-w (PMC9712435; doi:10.1038/s41467-022-35022-w)
Supplement: Supplementary file 1 — Supplementary Information [file 41467_2022_35022_MOESM1_ESM.docx]

Supplementary Information

[Supplementary Figures 2](#_Toc116858068)

[Supplementary Tables 19](#_Toc116858069)

# Supplementary Figures

**
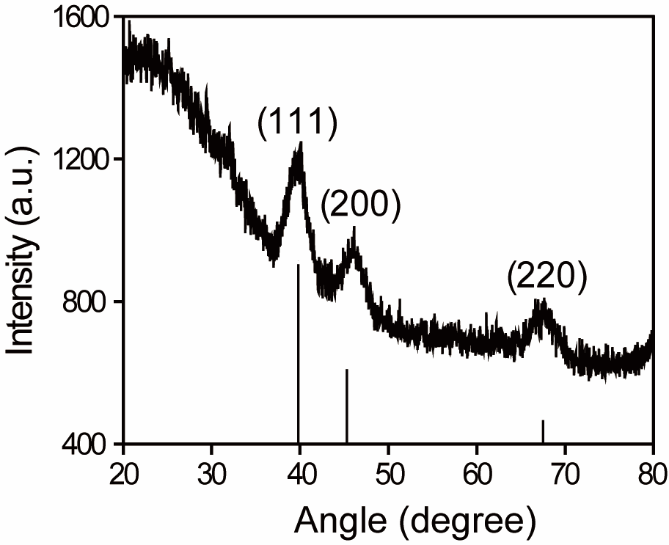
**

**Supplementary Figure 1. XRD pattern of PtNCs**. The XRD pattern shows the (111), (200), and (220) pattern of PtNCs (JCPDS No. 04-0802).


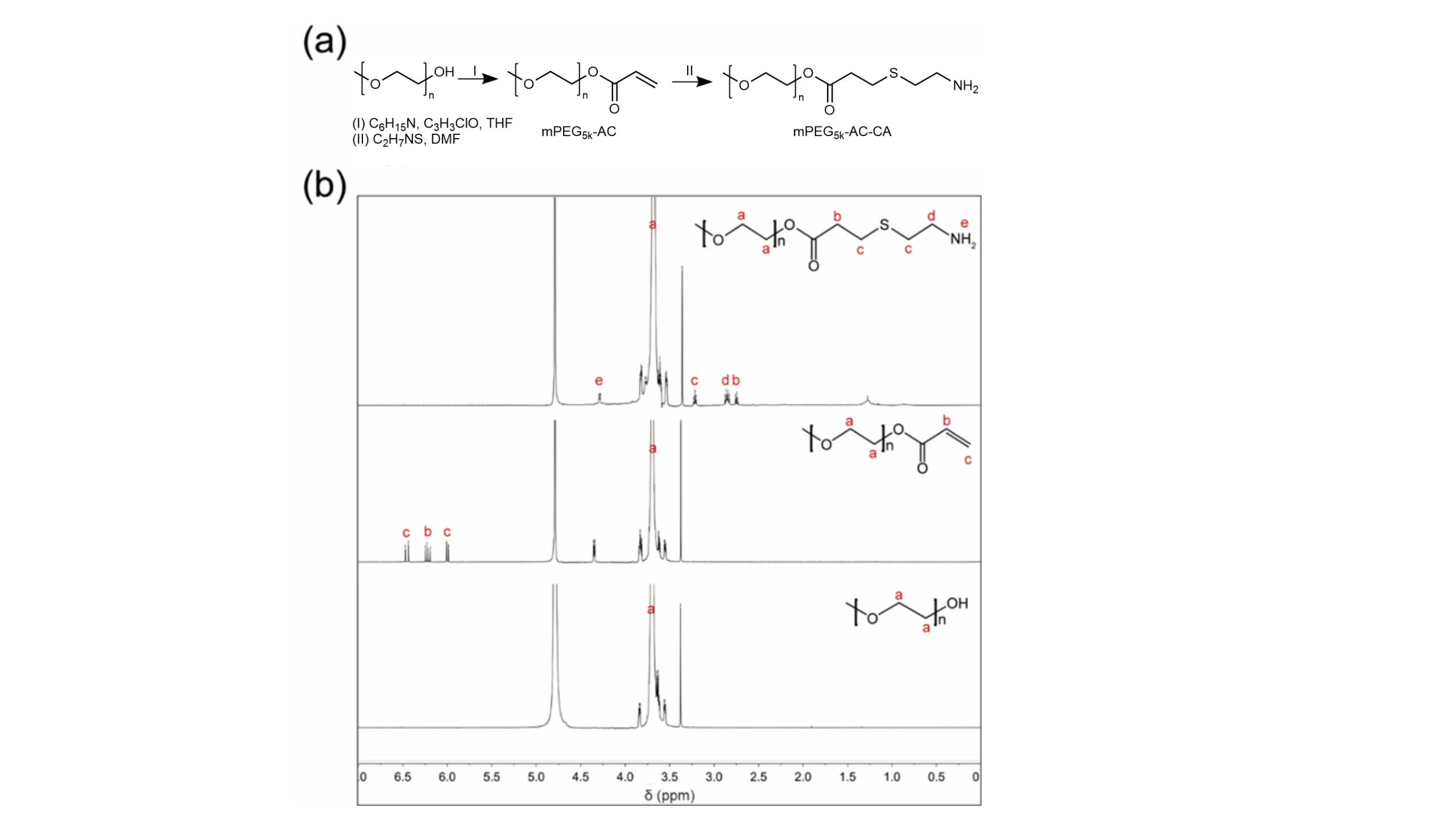


**Supplementary Figure 2. Synthesis and characterization of mPEG_5k_-OH, mPEG_5k_-AC, mPEG_5k_-AC-CA. a**, Synthetic scheme of pH-sensitive β-thiopropionate linker equipped PEG ligand (mPEG_5k_-AC-CA). **b** ^1^H-NMR analysis (500 MHz, D_2_O) of mPEG_5k_-OH, mPEG_5k_-AC, mPEG_5k_-AC-CA.


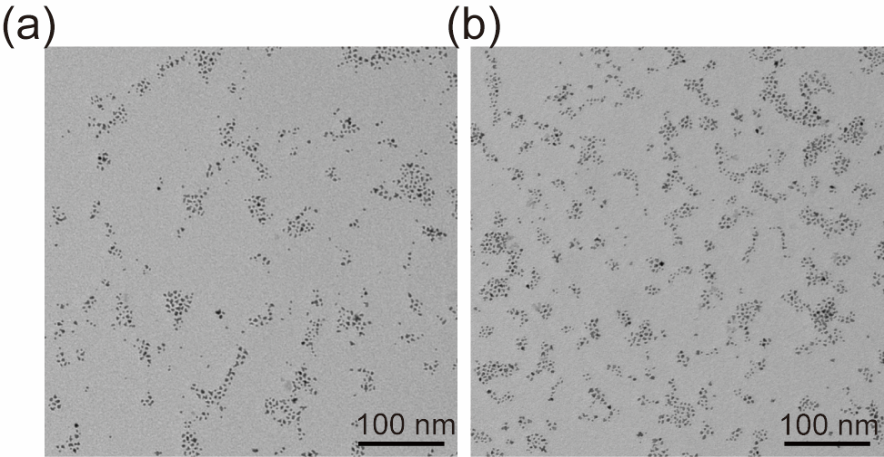


**Supplementary Figure 3.** **TEM images of PtNC@PEG and PtNC@TAT. a, b,** TEM images of PtNC@PEG (**a**) and PtNC@TAT (**b**). n = 3 independent experiments.


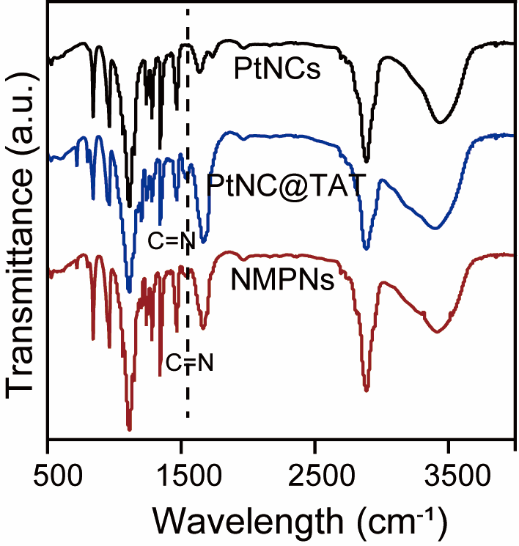


**Supplementary Figure 4. Characterization of surface-modification of PtNCs, PtNC@TAT, and NMPNs.** FT-IR spectrum of PtNCs, PtNC@TAT and NMPNs.


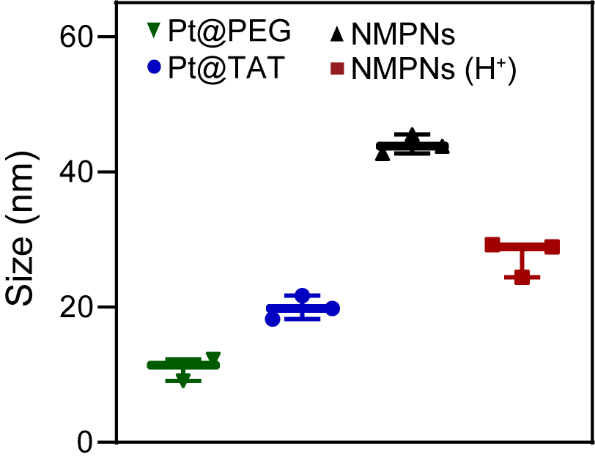


**Supplementary Figure 5.** **The sizes of PtNC@PEG, PtNC@TAT, and NMPNs.** The hydrodynamic diameters of PtNC@PEG (pH 7.4), PtNC@TAT (pH 7.4), NMPNs (pH 7.4) and NMPNs (pH 6.5). n = 3 independent experiments, data are presented as mean values ± SEM. Source data are provided as a Source Data file.


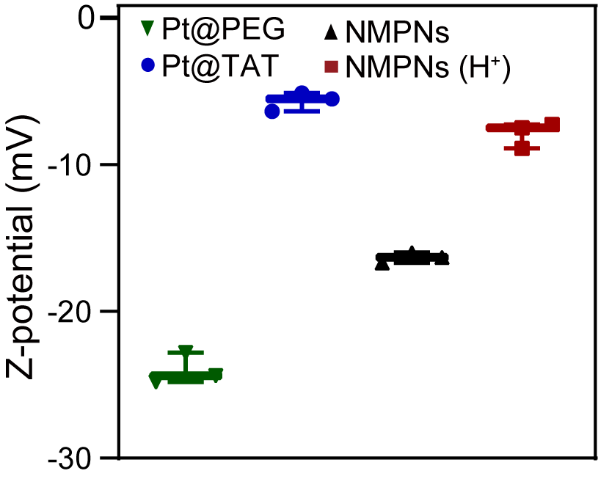


**Supplementary Figure 6.** **The zeta potentials of PtNC@PEG, PtNC@TAT, and NMPNs.** Zeta potentials of PtNC@PEG (pH 7.4), PtNC@TAT (pH 7.4), NMPNs (pH 7.4) and NMPNs (pH 6.5). n = 3 independent experiments, data are presented as mean values ± SEM. Source data are provided as a Source Data file.


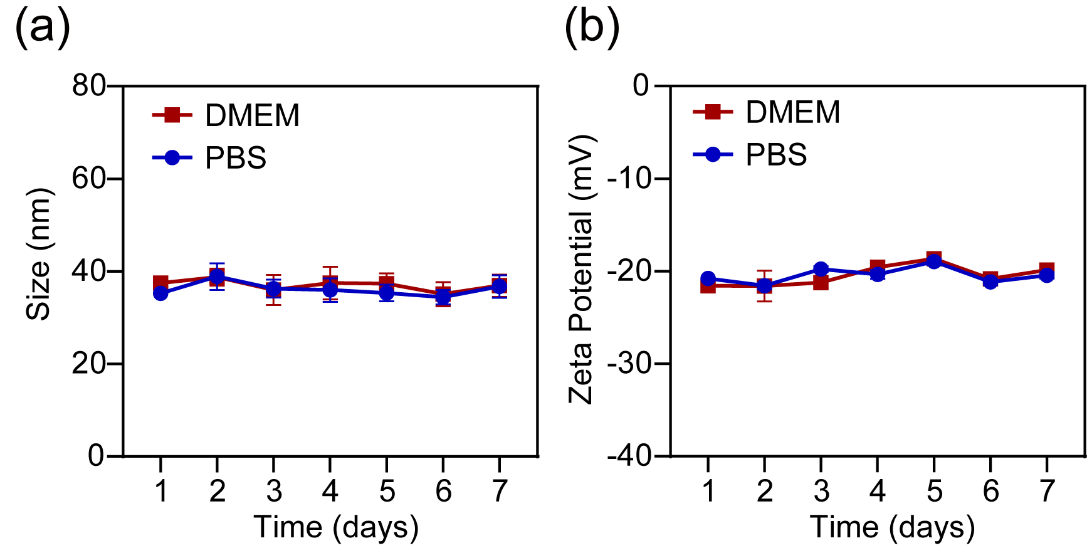


**Supplementary Figure 7.** **Characterization of the sizes and zeta potentials of NMPNs in DMEM or PBS.** **a, b,** The sizes (**a**) and zeta potentials (**b**) of NMPNs over a week. n = 3 independent experiments, data are presented as means ± S.E.M. Source data are provided as a Source Data file.


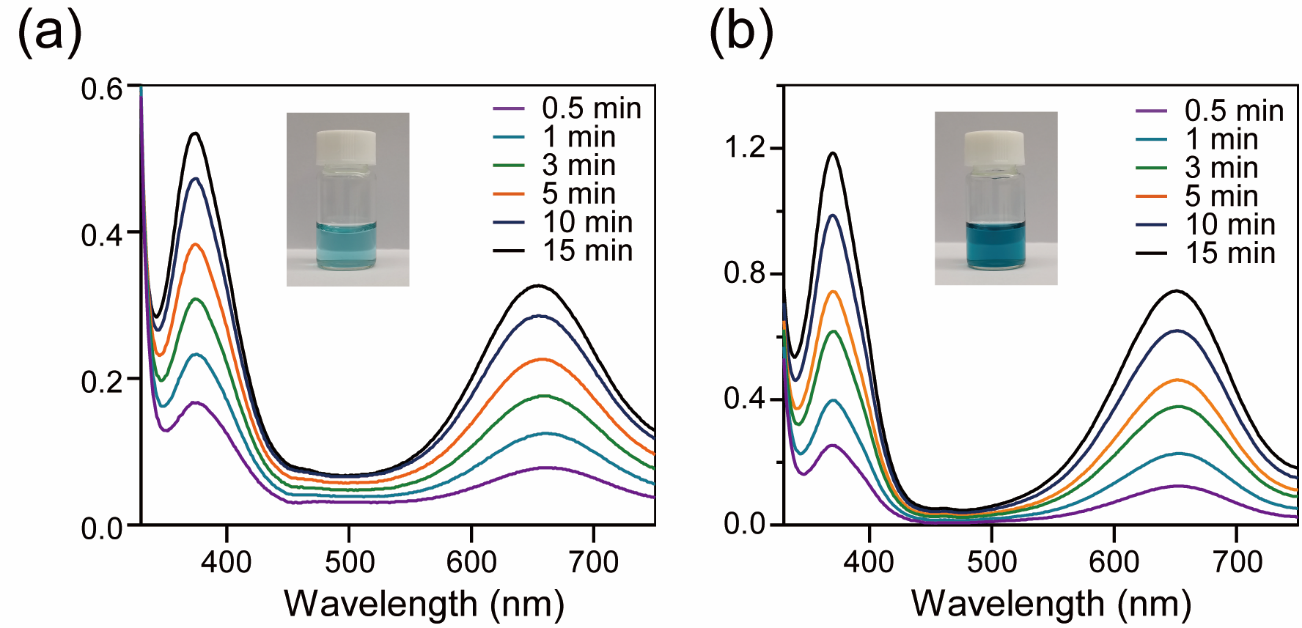


**Supplementary Figure 8.** **NMPNs induce TMB oxidation in the presence or absence of H_2_O_2_. a**, UV-vis absorption spectra of TMB oxidation induced by NMPNs at pH 7.4. **b**, UV-vis absorption spectra of TMB oxidation induced by NMPNs at pH 7.4 in the presence of H_2_O_2_ after purging with Ar.


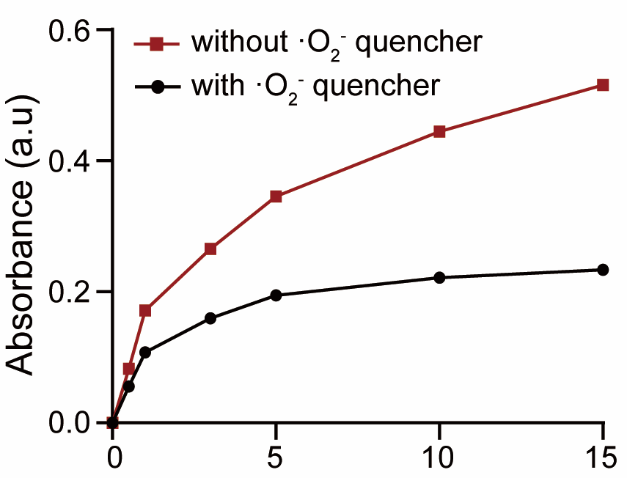


**Supplementary Figure 9. NMPNs induce TMB oxidation by generating**·**O_2_^-^.** Time-dependent absorbance of TMB oxidation product at 652 nm induced by NMPNs at pH 7.4 with or without the ·O_2_^-^ quencher.


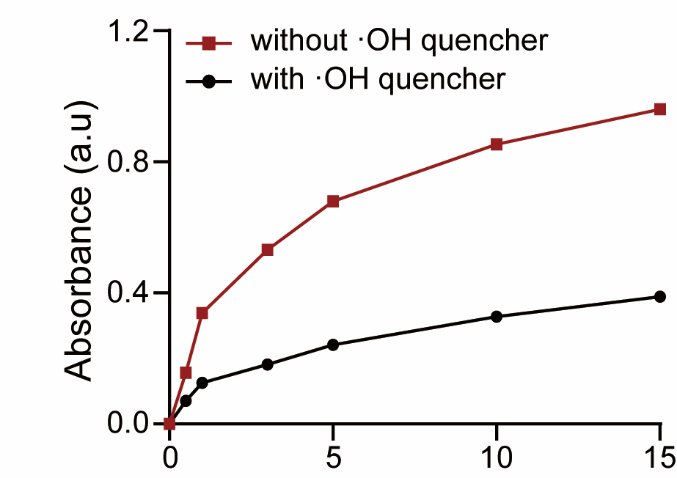


**Supplementary Figure 10. NMPNs induce TMB oxidation by generating ·OH.** Time-dependent absorbance of TMB oxidation product at 652 nm induced by NMPNs at pH 7.4 in the presence of H_2_O_2_ with or without the ·OH quencher.


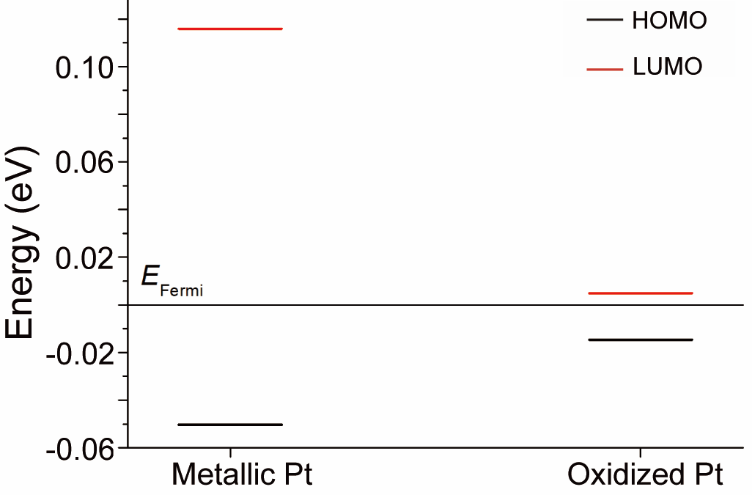


**Supplementary Figure 11. HOMO/LUMO potential energy of metallic Pt and oxidized Pt.**


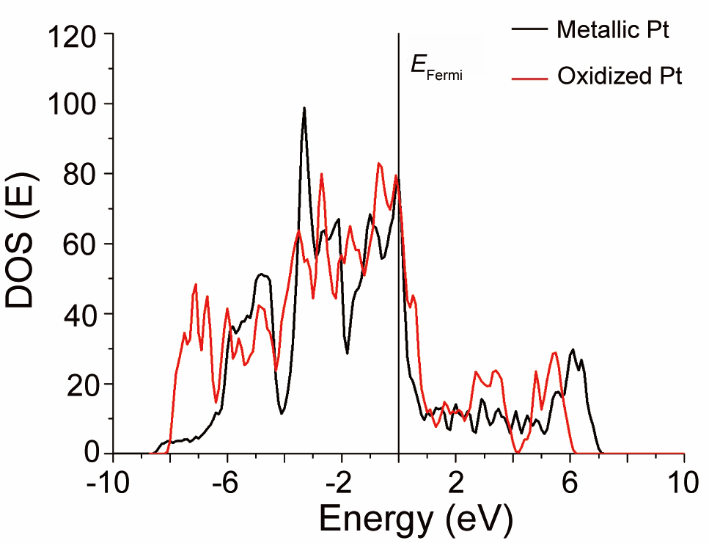


**Supplementary Figure 12. Density of states functions of metallic Pt and oxidized Pt.**


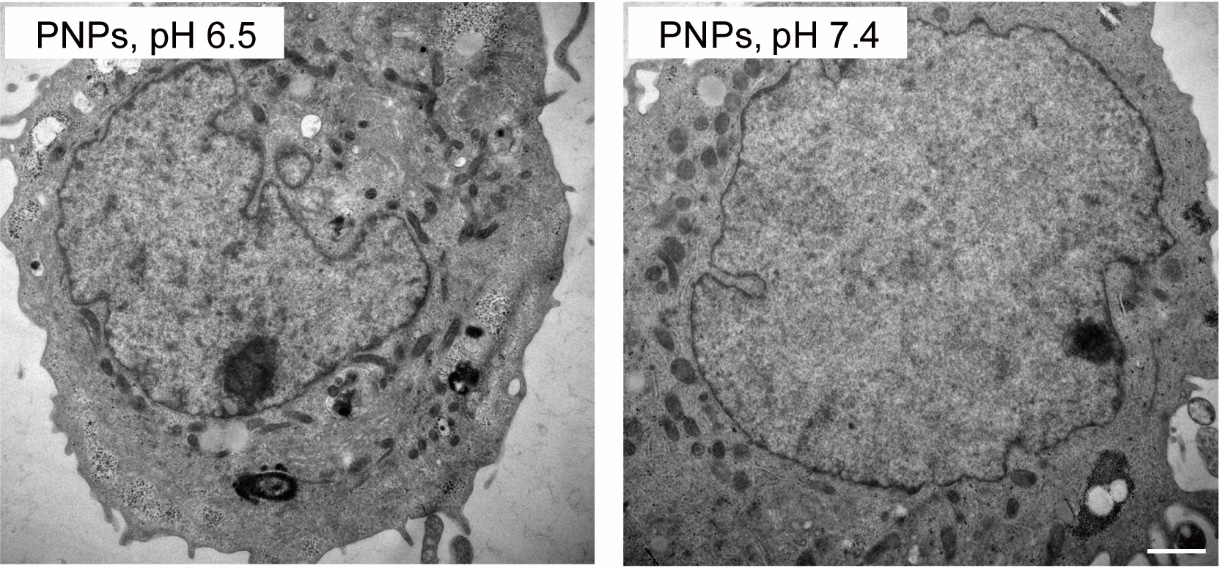


**Supplementary Figure 13. Bio-TEM images of cisplatin-resistant Huh7 cells treated by PNPs at different pH conditions.** Bio-TEM images of cells after incubation for 12 h with PNPs under different pH conditions. Scale bar: 1 μm. n = 3 independent experiments.


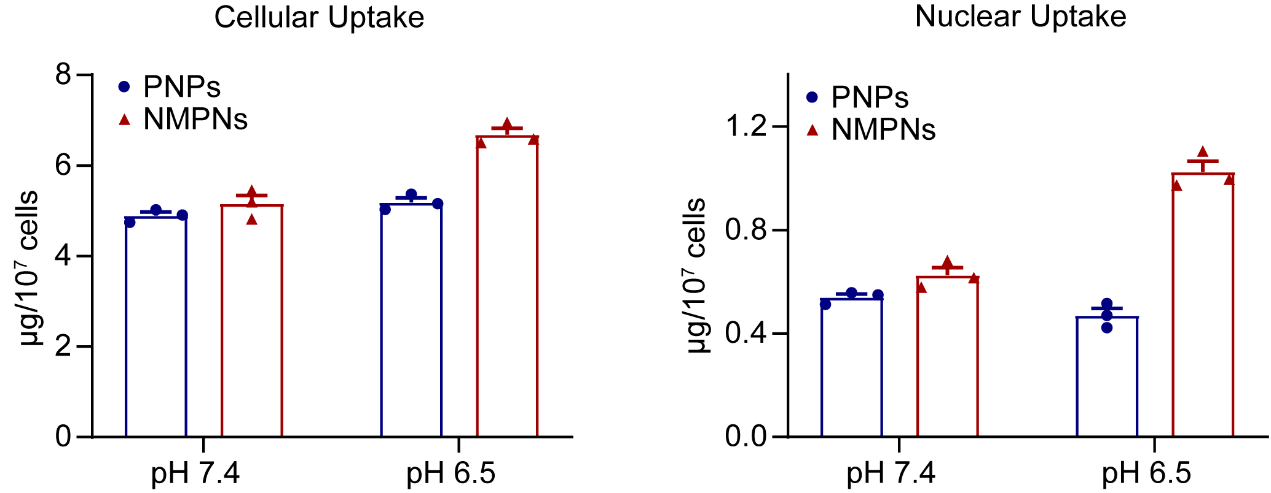


**Supplementary Figure 14.** **The concentrations of Pt ions in the nucleus of cisplatin-resistant Huh7 cells after treatment with NMPNS or PNPs under different pH conditions.** NMPNs can induce enhanced accumulation of Pt ions in the nucleus of cisplatin-resistance Huh7 cells at pH 6.5. n = 3 independent experiments, data are presented as means ± S.E.M. Source data are provided as a Source Data file.


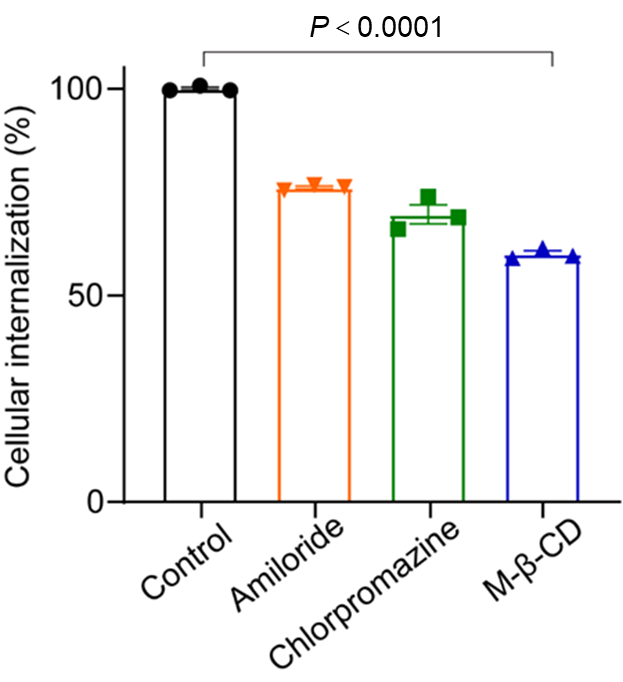


**Supplementary Figure 15. Characterization of the cellular endocytosis mechanism of NMPNs in cisplatin-resistant Huh7 cells.** Flow cytometry analysis of internalization of NMPNs into Huh7 cells after pre-treatment with serum-free medium (vehicle), amiloride (an inhibitor of macropinocytosis), chlorpromazine (an inhibitor of clathrin-mediated endocytosis), or methyl-b-cyclodextrin (MβCD, an inhibitor of caveolin-mediated endocytosis). n = 3 independent experiments, data are presented as means ± S.E.M. Statistical significance was analyzed by one-way ANOVA. Source data are provided as a Source Data file.


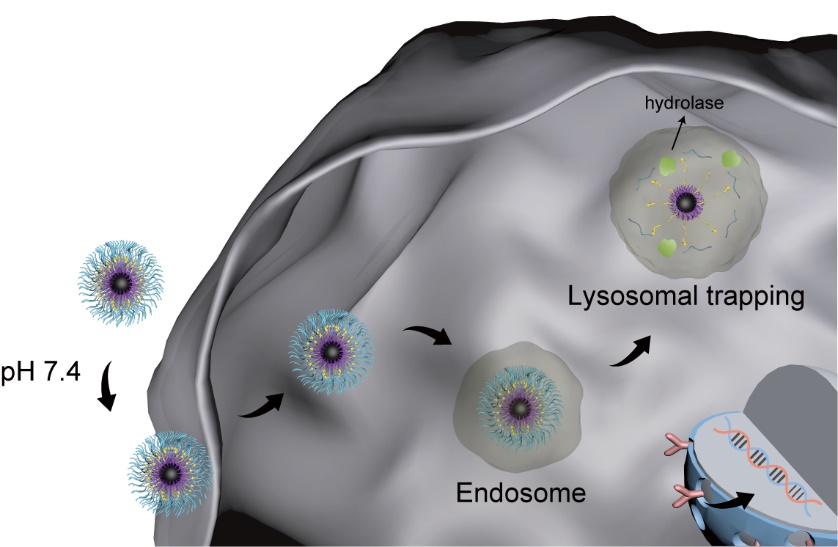


**Supplementary Figure 16. Schematic diagram of the cellular process of PNPs under neutral conditions.** NMPNs implement the pH-responsive discharge of the “protective shield” under acidic conditions to exposure TAT peptides for facilitating the endosomal escape and subsequent nucleus targeting.


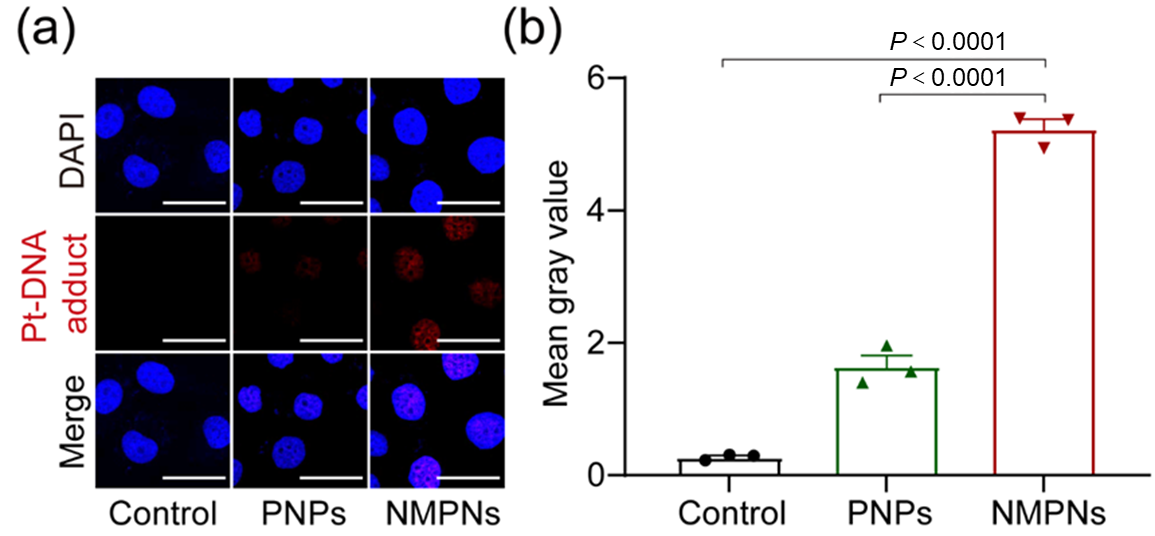


**Supplementary Figure 17. NMPNs can significantly induce the formation of Pt-DNA adducts. a**, Immunofluorescence images of the Pt-DNA adducts in cisplatin-resistant Huh7 cells after different treatments at pH 6.5. Scale bar: 40 μm. **b**, Quantification analysis of Pt-DNA adducts in cisplatin-resistant Huh7 cells after different treatments at pH 6.5. n = 3 independent experiments, data are presented as means ± S.E.M. Statistical significance was analyzed by one-way ANOVA. Source data are provided as a Source Data file.


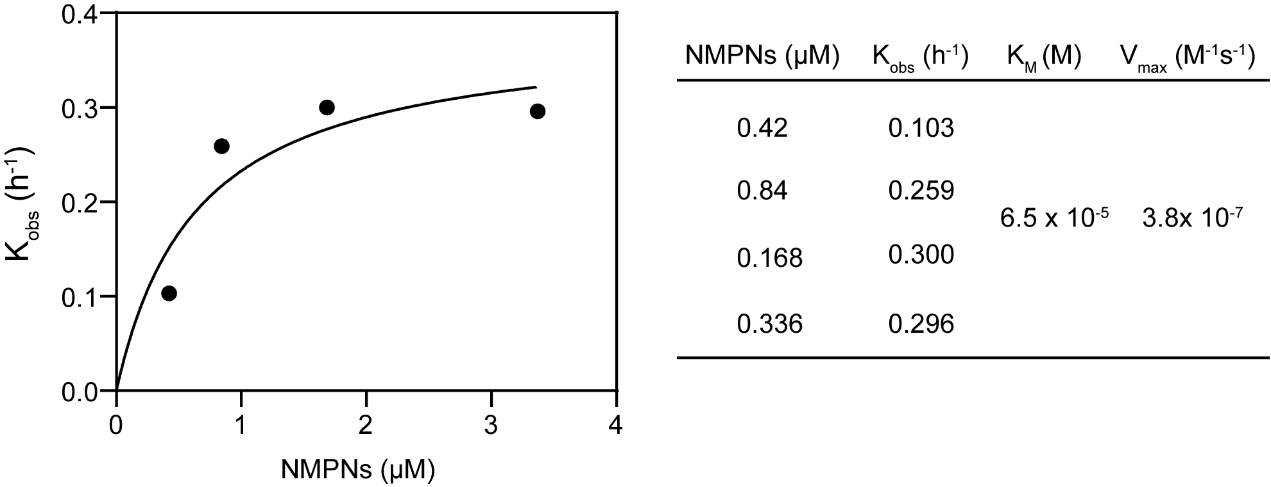


**Supplementary Figure 18.** **Pseudo Michaelis-Menten kinetics of the cleavage of DNA (∼300 ng) after treatment with NMPNs.** The pseudo Michaelis-Menten kinetic parameters *V_max_* and *K_M_* were calculated to be 3.8 × 10^−7^ M ^−1^s^−1^ and 6.5 × 10^−5^ M, respectively.


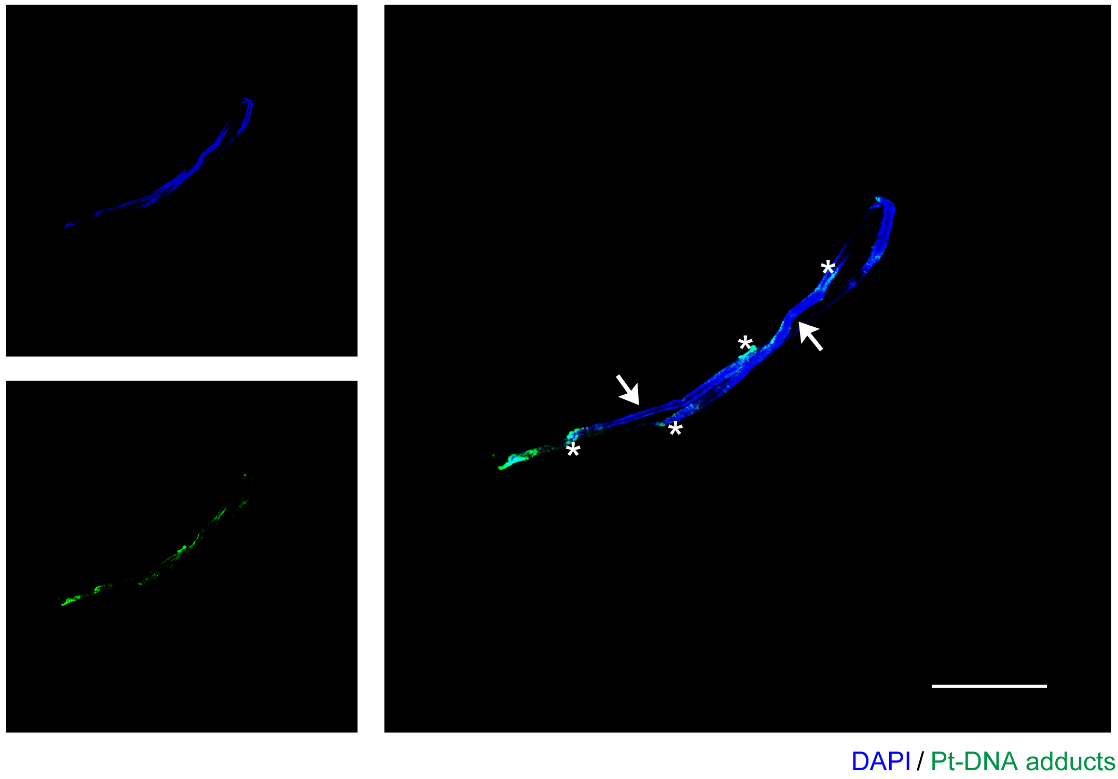


**Supplementary Figure 19.** **The analysis of Pt-DNA binding sites in the DNA extracted from cisplatin-resistant Huh7 cells after treatment with NMPNs.** Arrows indicate the DNA fragmentations. Asterisks indicate the Pt-DNA binding sites at the end of DNA fragmentations. n = 3 independent experiments. Scale bar: 100 μm.


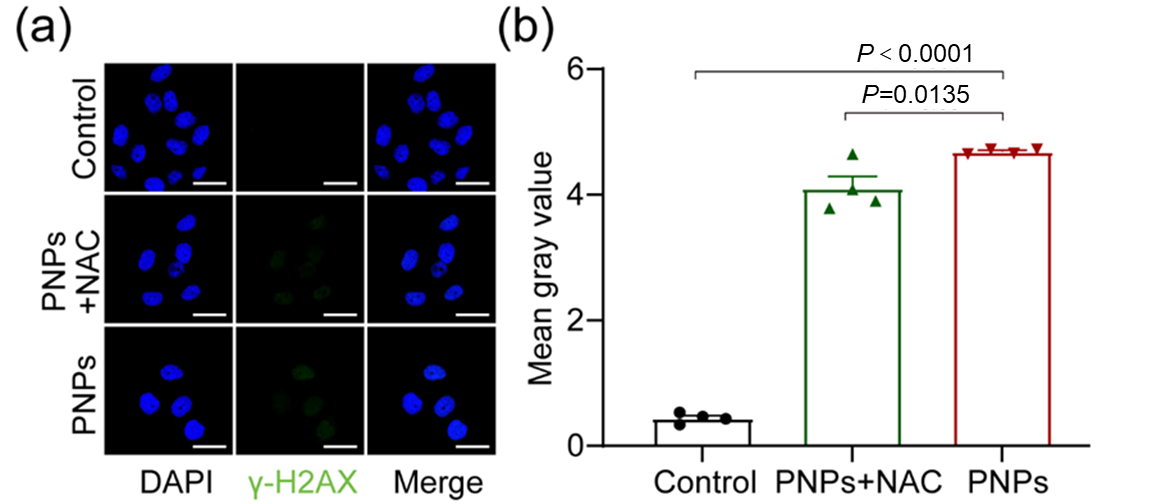


**Supplementary Figure 20**. **The co-treatment of NAC shows no interference with the capability of PNPs to induce Pt-DNA adducts formation. a**, Immunofluorescence images of the γ-H2AX in cisplatin-resistant Huh7 cells after treatments with PNPs with or without NAC at pH 6.5. Scale bar: 40 μm. **b**, Quantification analysis of Pt-DNA adducts in cisplatin-resistant Huh7 cells after treatments with PNPs with or without NAC at pH 6.5. n = 4 independent experiments, data are presented as means ± S.E.M. Statistical significance was analyzed by one-way ANOVA with multiple comparisons test. Source data are provided as a Source Data file.


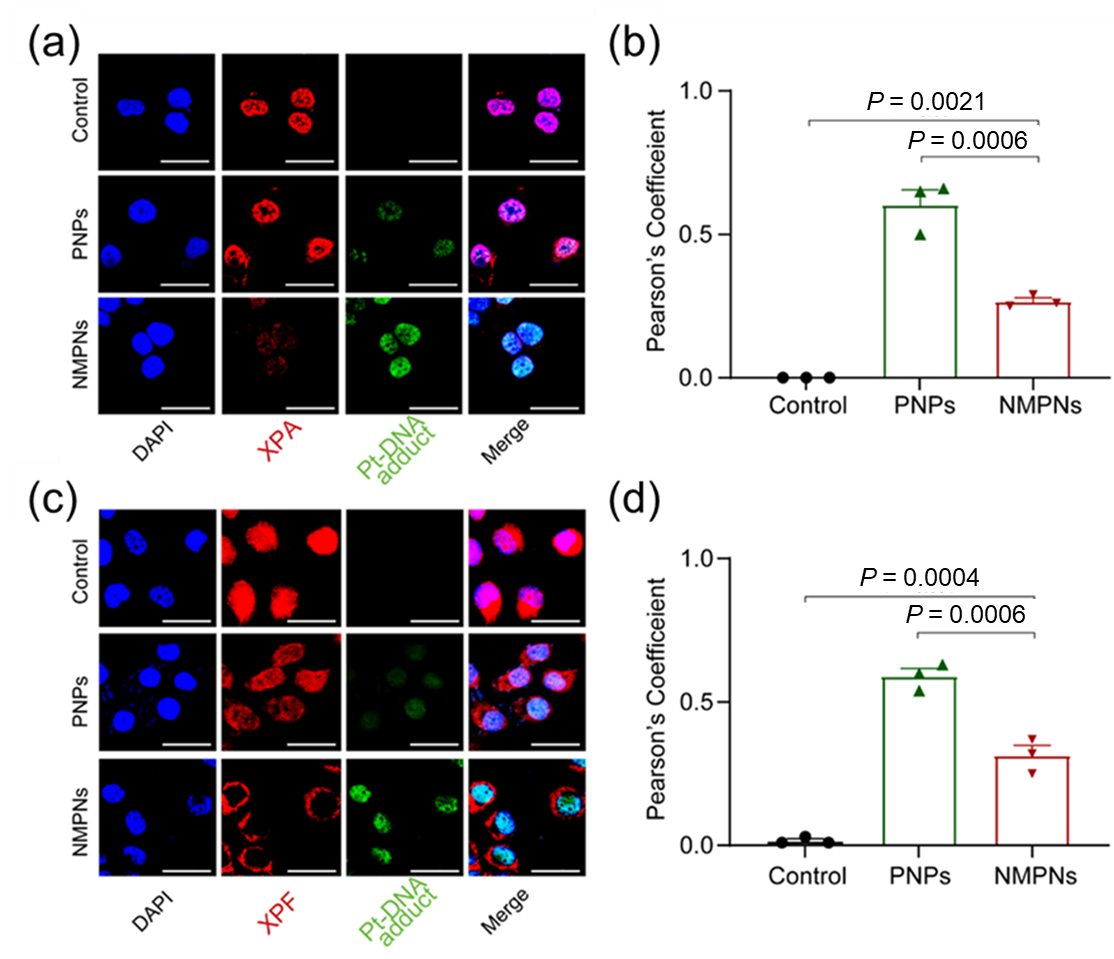


**Supplementary Figure 21. NMPNs can induce DNA damage that cannot be repaired by NER pathway. a**, Immunofluorescence images of the XPA and Pt-DNA adducts in cisplatin-resistant Huh7 cells after treatment with PNPs or NMPNs at pH 6.5 for 24 h. Scale bar: 40 μm. **b** Quantification analysis of the colocalization between XPA and Pt-DNA adducts. **c**, Immunofluorescence images of the XPF and Pt-DNA adducts in cisplatin-resistant Huh7 cells after treatment with PNPs or NMPNs at pH 6.5 for 24 h. Scale bar: 40 μm. **d** Quantification analysis of the colocalization between XPF and Pt-DNA adducts. Scale bar: 40 μm. n = 3 independent experiments, data are presented as means ± S.E.M. Statistical significance was analyzed by one-way ANOVA with multiple comparisons test. Source data are provided as a Source Data file.


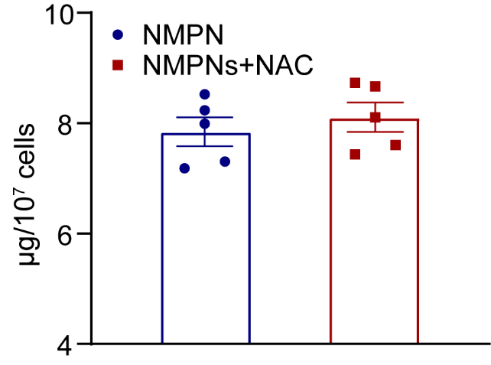


**Supplementary Figure 22. The co-treatment of NAC shows no interference with the cellular uptake of NMPNs.** The concentrations of Pt ions in the cisplatin-resistant Huh7 cells after treatment with NMPNs or NMPNs+NAC. n = 5 independent experiments, data are presented as means ± S.E.M. Statistical significance was analyzed by two-tailed Student’s t-test. Source data are provided as a Source Data file.

**
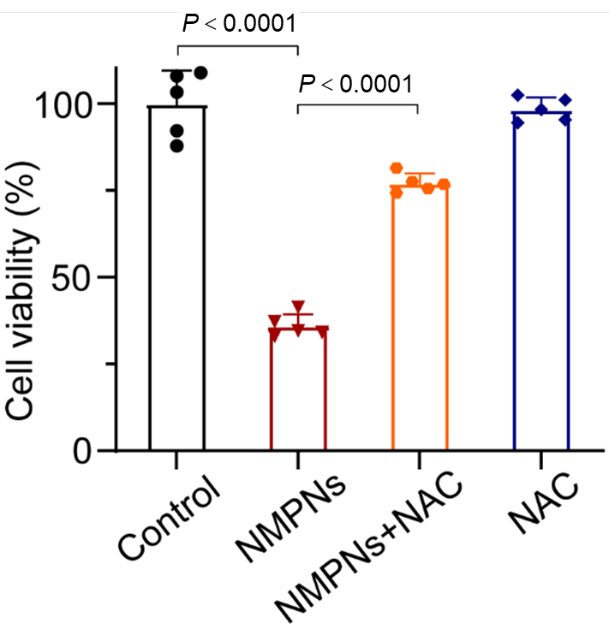
**

**Supplementary Figure 23.** **The co-treatment of NAC can impair the inhibition effect of NMPNs on cisplatin-ressistant Huh7 cells.** The inhibition effect of saline, NMPNs, NAC, or NMPNs+NAC on cisplatin-resistant Huh7 cells growth. n = 5 independent experiments, data are presented as means ± S.E.M. Statistical significance was analyzed by one-way ANOVA with multiple comparisons test. Source data are provided as a Source Data file.


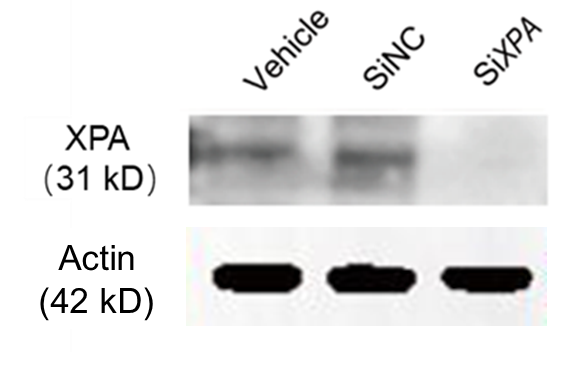


**Supplementary Figure 24.** **The XPA expression in cisplatin-resistant Huh7 cells after different treatments.** Western blot analysis of XPA expression in cisplatin-resistant Huh7 cells, siNC-transfected cisplatin-resistant Huh7 cells and si*XPA*-transfected cisplatin-resistant Huh7 cells, respectively. n = 3 independent experiments. Source data are provided as a Source Data file.


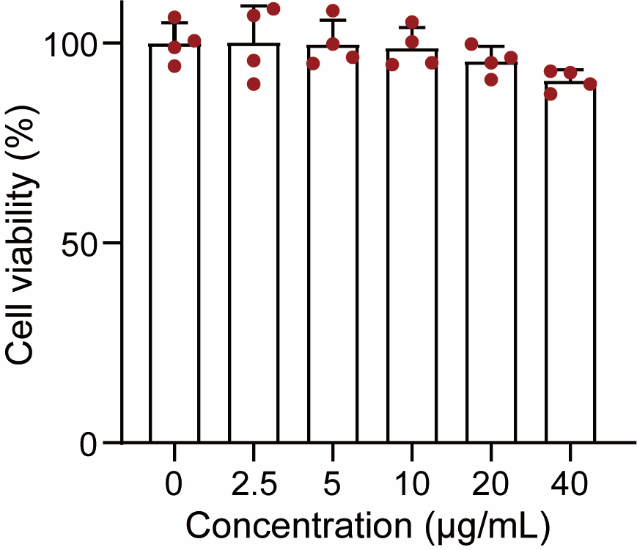


**Supplementary Figure 25.** **NMPNs show no obvious toxicity to L02 cells.** Cell viability of L02 cell lines after NMPNs treatment in the neutral condition. n = 4 independent experiments, data are presented as means ± S.E.M. Source data are provided as a Source Data file.


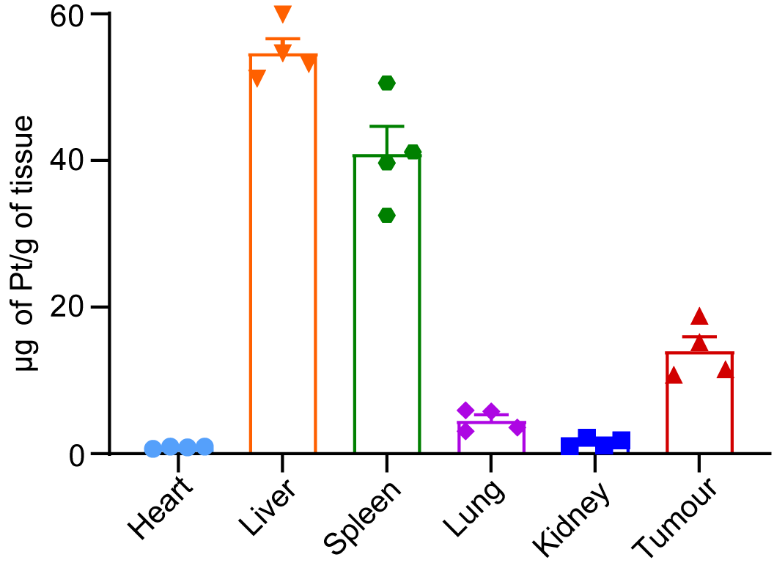


**Supplementary Figure 26.** **Biodistribution of NMPNs in mice treated with NMPNs via ICP-MS analysis.** NMPNs can effectively accumulates in the tumor tissues. n = 4 independent experiments, data are presented as means ± S.E.M. Source data are provided as a Source Data file.


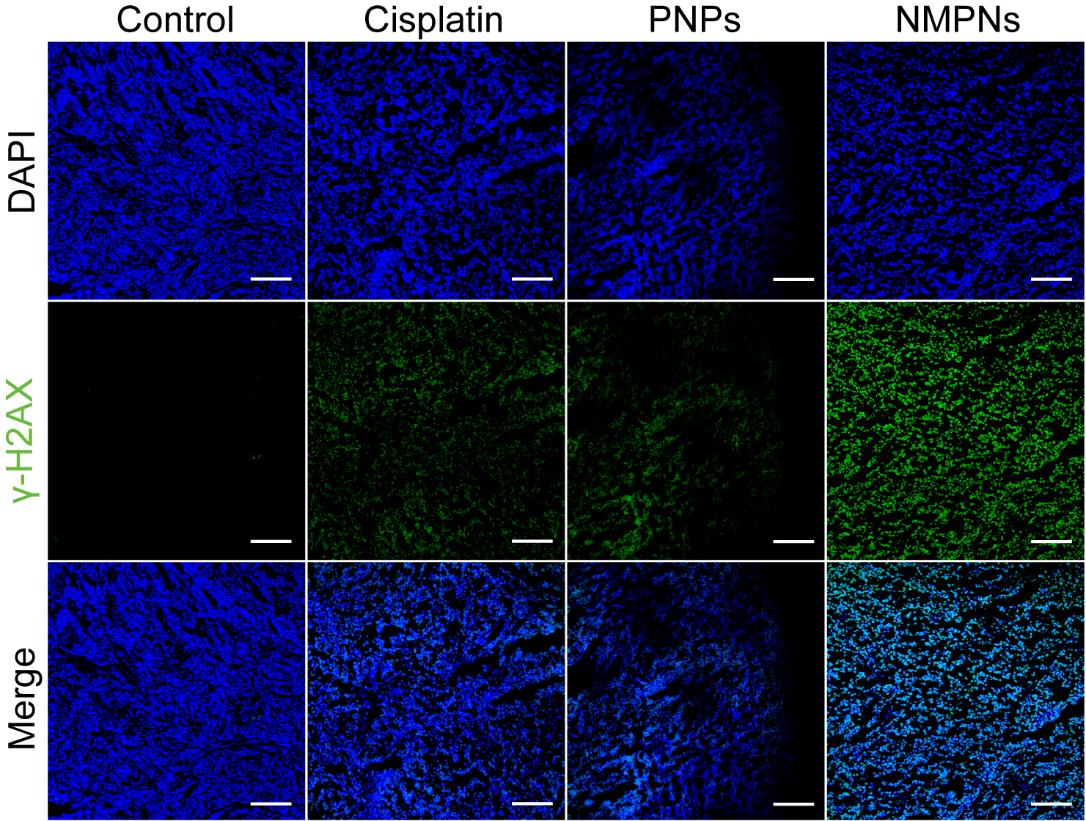


**Supplementary Figure 27. Immunofluorescence images of γ-H2AX in the tumour tissues after different treatments.** NMPNs treatment can significantly induce DNA damage as compared to the treatment with cisplatin or PNPs. n = 3 independent mice. Scale bar: 200 μm.


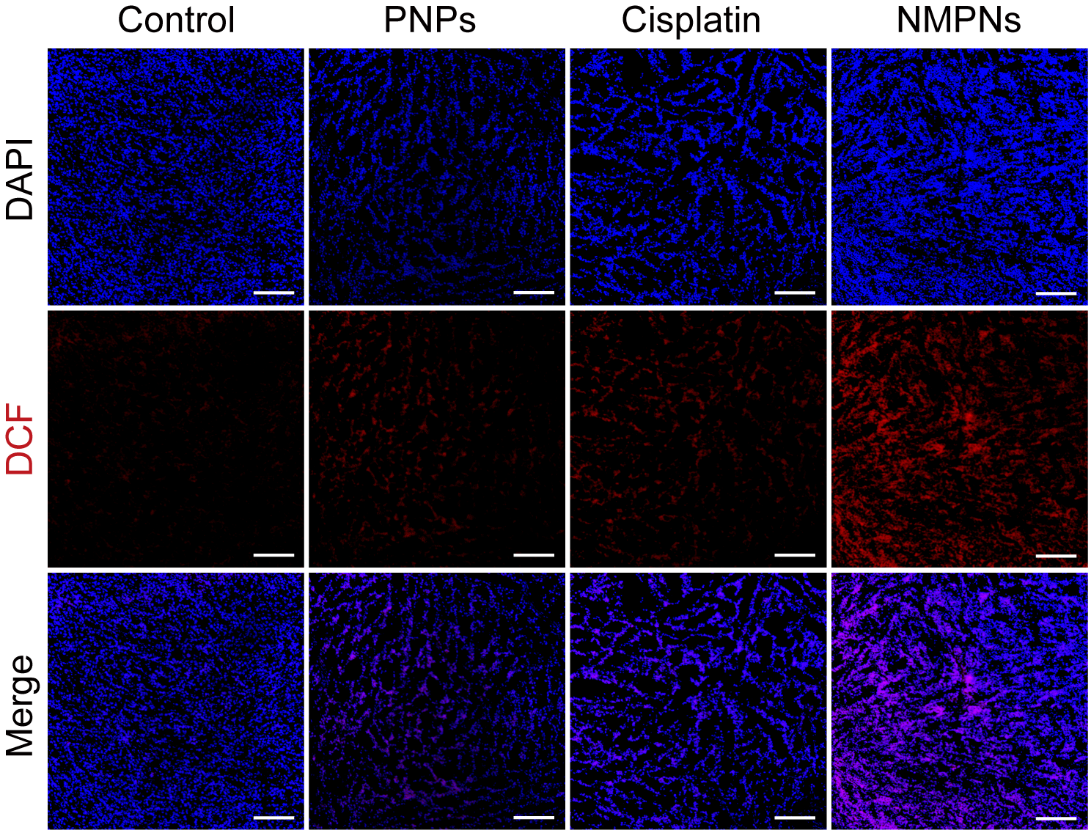


**Supplementary Figure 28.** **The levels of ROS in tumour tissues after treatment with cisplatin, PNPs or NMPNs.** NMPNs treatment can profoundly enhance the ROS level in the tumour tissues as compared to the treatment with cisplatin and PNPs. n = 3 independent mice. Scale bar: 200 μm.


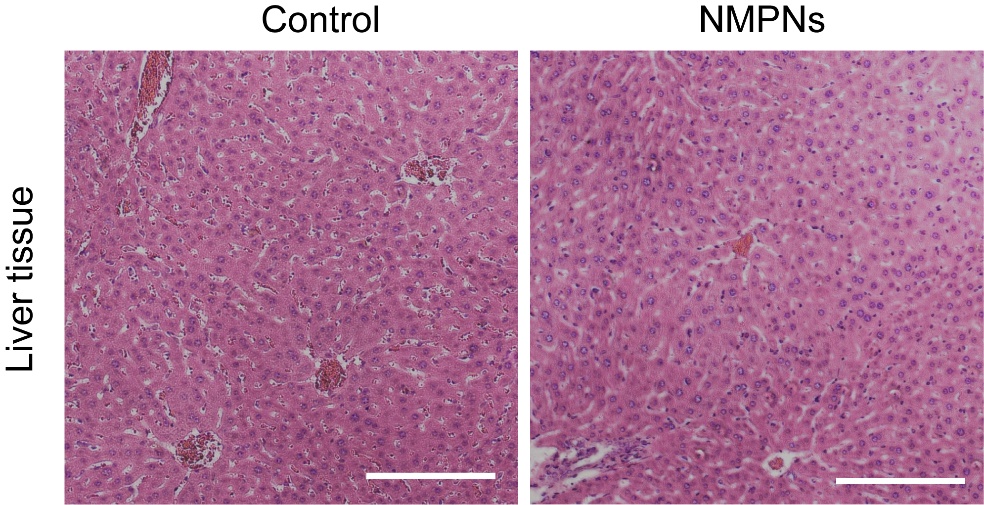


**Supplementary Figure 29. Hematoxylin and eosin (H&E) staining images of normal tissues around tumour in the liver after different treatments.** Representative H&E staining images of normal tissues around tumour in the liver from saline or NMPNs-treated mice. n = 3 independent mouse livers. Scale bar: 500 μm.


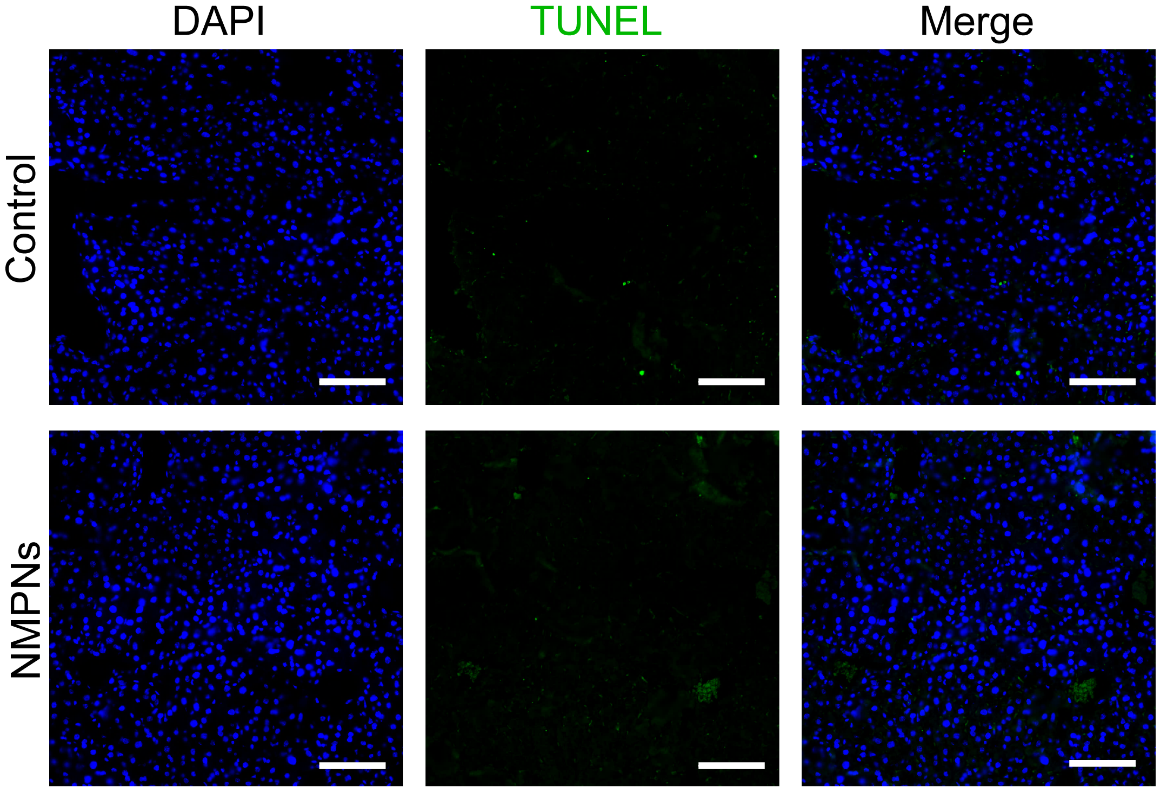


**Supplementary Figure 30.** **TUNEL staining images of normal tissues around tumour in the liver after different treatments.** Representative TUNEL staining images of normal tissues around tumour in the liver from saline or NMPNs-treated mice. n = 3 independent mouse livers. Scale bar: 100 μm.


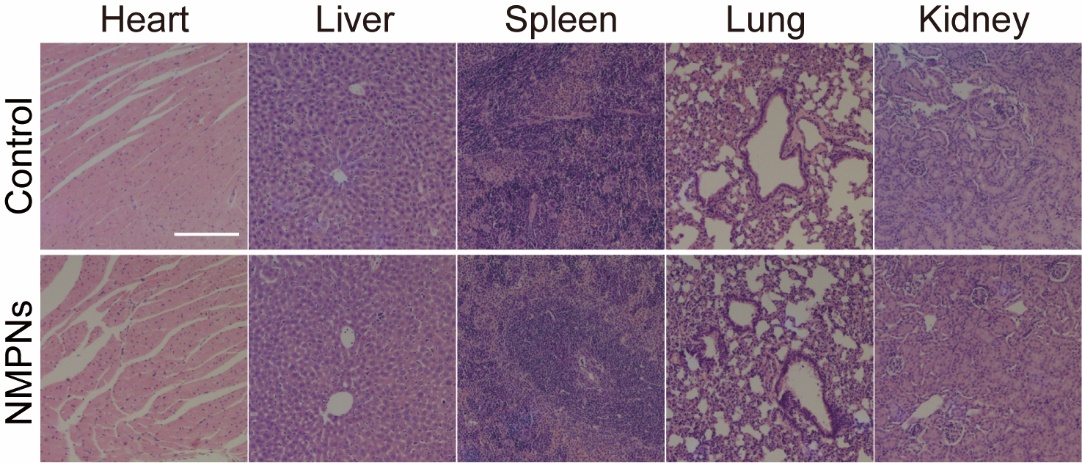


**Supplementary Figure 31.** **Histopathological examination of mice organs**. These results show no toxicity of NMPNs to the main organs of mice. n = 3 independent mice. Scale bar: 500 μm.


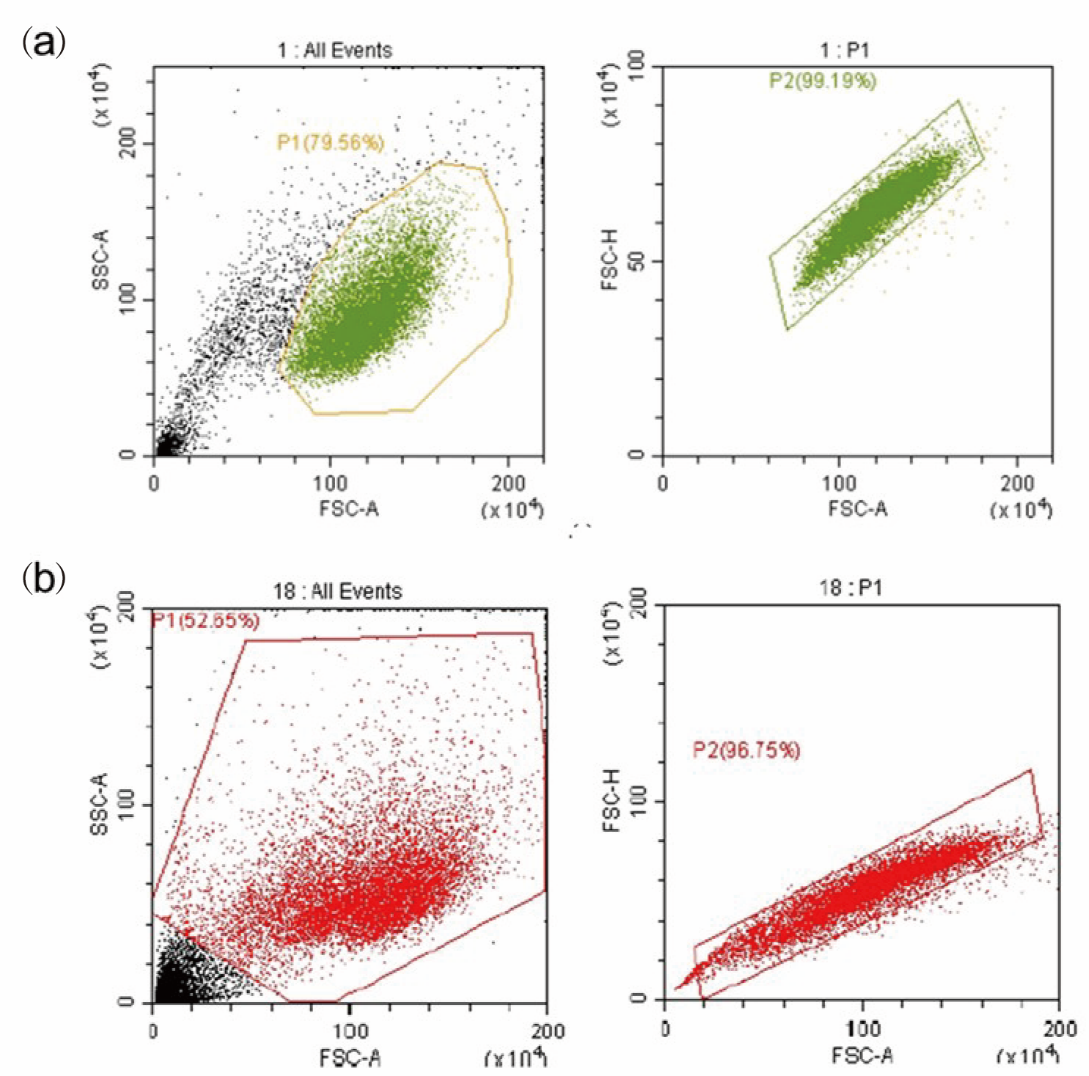


**Supplementary Figure 32.** **Gating strategies of flow cytometry.** **a**, Gating strategy to determine the internalization of NMPNs into Huh7 cells presented in Supplementary Fig. 15. **b**, Gating strategy to determine the percentage of Annexin V-FITC^+^ PI^+^ cells and Annexin V-FITC^+^PI^-^ cells presented in Fig. 5e, f.

# Supplementary Tables


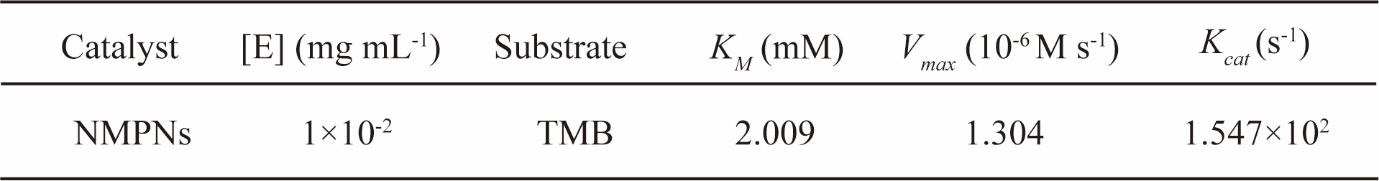


**Supplementary Table 1.** The Michaelis-Menten constant (*K_M_)* and maximum reaction rate (*V_max_*) of NMPNs with TMB as the substrate for OXD-like catalysis. Source data are provided as a Source Data file.


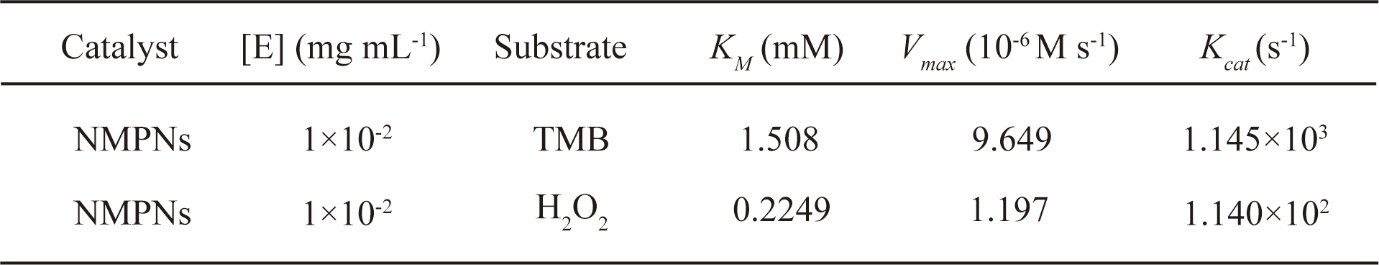


**Supplementary Table 2.** The *K_M_* and *V_max_* of NMPNs with H_2_O_2_ and TMB as the substrates for POD-like catalysis. Source data are provided as a Source Data file.
