# Supplementary material for: A nuclease-mimetic platinum nanozyme induces concurrent DNA platination and oxidative cleavage to overcome cancer drug resistance
Source: Nat Commun. 2022 Nov 30;13:7361. doi: 10.1038/s41467-022-35022-w (PMC9712435; doi:10.1038/s41467-022-35022-w)
Supplement: Supplementary file 3 — Reporting Summary [file 41467_2022_35022_MOESM3_ESM.pdf]

nature portfolio

Corresponding author(s): Daishun Ling

Last updated by author(s): Oct 18, 2022

Reporting Summary

Nature Portfolio wishes to improve the reproducibility of the work that we publish. This form provides structure for consistency and transparency in reporting. For further information on Nature Portfolio policies, see our Editorial Policies and the Editorial Policy Checklist.

Statistics

For all statistical analyses, confirm that the following items are present in the figure legend, table legend, main text, or Methods section.

n/a	Confirmed	
☐	☒	The exact sample size (*n*) for each experimental group/condition, given as a discrete number and unit of measurement
☐	☒	A statement on whether measurements were taken from distinct samples or whether the same sample was measured repeatedly
☐	☒	The statistical test(s) used AND whether they are one- or two-sided<br>*Only common tests should be described solely by name; describe more complex techniques in the Methods section.*
☒	☐	A description of all covariates tested
☒	☐	A description of any assumptions or corrections, such as tests of normality and adjustment for multiple comparisons
☐	☒	A full description of the statistical parameters including central tendency (e.g. means) or other basic estimates (e.g. regression coefficient) AND variation (e.g. standard deviation) or associated estimates of uncertainty (e.g. confidence intervals)
☐	☒	For null hypothesis testing, the test statistic (e.g. *F*, *t*, *r*) with confidence intervals, effect sizes, degrees of freedom and *P* value noted<br>*Give P values as exact values whenever suitable.*
☒	☐	For Bayesian analysis, information on the choice of priors and Markov chain Monte Carlo settings
☒	☐	For hierarchical and complex designs, identification of the appropriate level for tests and full reporting of outcomes
☒	☐	Estimates of effect sizes (e.g. Cohen's *d*, Pearson's *r*), indicating how they were calculated

Our web collection on statistics for biologists contains articles on many of the points above.

Software and code

Policy information about availability of computer code

Data collection	The catalytic activities was recorded by using the Bruker A300 EPR spectrometer. The quantification of NMPNs in cell and tissues was analyzed by using ICP-MS (Agilent Technologies 7800). Western blot was carried out using enhanced chemiluminescence (ECL) detection reagents (FDbio Science Biotech Co., Ltd., Hangzhou, China). Flow cytometry was performed using fluorescence microscope (CytoFLEX LX, USA). Characterization of nanomaterials properties were carried out from transmission electron microscopy (Hitachi HT7700, Japan), XPS (Thermo Scientific ESCALAB 250 Xi, UK) and XRD (PANalytical B.V. X-pert Powder, the Netherlands).
Data analysis	Statistical comparisons were performed using Bruker WinEPR Acquisition (version 4.40), Graphpad Prism (version 8.0) and Image J (version 1.8.0)

For manuscripts utilizing custom algorithms or software that are central to the research but not yet described in published literature, software must be made available to editors and reviewers. We strongly encourage code deposition in a community repository (e.g. GitHub). See the Nature Portfolio guidelines for submitting code & software for further information.

Data

Policy information about availability of data

All manuscripts must include a data availability statement. This statement should provide the following information, where applicable:

- Accession codes, unique identifiers, or web links for publicly available datasets
- A description of any restrictions on data availability
- For clinical datasets or third party data, please ensure that the statement adheres to our policy

The authors declare that all data from this study are available in the paper and its Supplementary Information. Raw data for the individual measurements are available on reasonable request. This paper uses only the Protein Data Bank (PDB ID: 6WQG [https://www.rcsb.org/structure/6WQG]) which has been mentioned in the Methods section.

Human research participants

Policy information about studies involving human research participants and Sex and Gender in Research.

Reporting on sex and gender	Not applicable
Population characteristics	Not applicable
Recruitment	Not applicable
Ethics oversight	Not applicable

Note that full information on the approval of the study protocol must also be provided in the manuscript.

Field-specific reporting

Please select the one below that is the best fit for your research. If you are not sure, read the appropriate sections before making your selection.

[x] Life sciences [ ] Behavioural & social sciences [ ] Ecological, evolutionary & environmental sciences

For a reference copy of the document with all sections, see nature.com/documents/nr-reporting-summary-flat.pdf

Life sciences study design

All studies must disclose on these points even when the disclosure is negative.

Sample size	Sample size choice was based on previous studies (ref. He, X., Zhang, L., Queme, L. et al, 2018. https://doi.org/10.1038/nm.4483; Huang, J., Li, J., Lyu, Y. et al. https://doi.org/10.1038/s41563-019-0378-4; Ni, D., Jiang, D., Kutyreff, C.J. et al. Ni, D., Jiang, D., Kutyreff, C.J. et al. ; Jiang, D., Ge, Z., Im, HJ. et al. https://doi.org/10.1038/s41551-018-0317-8; Deng, H., Yang, W., Zhou, Z. et al. https://doi.org/10.1038/s41467-020-18745-6), not predetermined by a statistical method. Sample sizes were indicated in the legend of each Figure and Supplementary Figure.
Data exclusions	No data were excluded.
Replication	We confirm all attempts at replication were successful. The experiments of western blots were independently replicated at least twice, and other experimental findings were all replicated at least 3 times independently.
Randomization	All samples were randomly allocated into experimental groups.
Blinding	Investigators were not blinded for nanomaterial synthesis. For in vivo experiments, the investigators were blinded to group allocation during data collection and analysis.

Reporting for specific materials, systems and methods

We require information from authors about some types of materials, experimental systems and methods used in many studies. Here, indicate whether each material, system or method listed is relevant to your study. If you are not sure if a list item applies to your research, read the appropriate section before selecting a response.

Materials & experimental systems

n/a	Involved in the study
☐	☒ Antibodies
☐	☒ Eukaryotic cell lines
☒	☐ Palaeontology and archaeology
☐	☒ Animals and other organisms
☒	☐ Clinical data
☒	☐ Dual use research of concern

Methods

n/a	Involved in the study
☒	☐ ChIP-seq
☐	☒ Flow cytometry
☒	☐ MRI-based neuroimaging

Antibodies

Antibodies used	anti-XPA (1:100, AF5336) and anti-Actin (1:1000, AA128) from Beyotime Biotechnology (Shanghai, China); anti-XPF (1:100, OM201253) from Omnimabs (California, USA); anti cisplatin-modified DNA (dilution 1:100, GTX17412) from GeneTex (California, USA); anti-γ-H2AX (dilution 1:100, ab81299) from Abcam (Cambridge, UK); Alexan Fluor 488-conjugated rabbit anti-rat IgG (H+L) (dilution 1:200, BA1129), Alexa Fluor 488-conjugated goat anti-rabbit IgG (H+L) (dilution 1:200, BA1127) and Alexa Fluor 555-conjugated goat anti-mouse IgG (H+L) (dilution 1:200, BA1126) from Boster Biological Technology Co., Ltd.
Validation	anti-XPA antibody<br>https://www.beyotime.com/product/AF5336.htm<br><br>anti-Actin antibody<br>https://www.beyotime.com/product/AA128.htm<br><br>anti cisplatin modified DNA antibody<br>https://www.genetex.cn/Product/Detail/Cisplatin-modified-DNA-antibody-CP9-19/GTX17412<br><br>anti-XPF antibody<br>http://www.omnimabs.com/antibody_XPF_Antibody_H_300-OM201253.html<br><br>anti-γ-H2AX antibody<br>https://www.abcam.cn/gamma-h2ax-phospho-s139-antibody-ep8542y-ab81299.html

Eukaryotic cell lines

Policy information about cell lines and Sex and Gender in Research

Cell line source(s)	L02 cells (CL-0111) and Huh7 (CL-0120) were obtained from iCell Bioscience Inc. (Shanghai, China).
Authentication	Cells were identified by Short Tandem Repeat (STR) method.
Mycoplasma contamination	The cell line was tested negative for mycoplasm contamination per suppliers.
Commonly misidentified lines (See ICLAC register)	No commonly misidentified lines were used.

Animals and other research organisms

Policy information about studies involving animals; ARRIVE guidelines recommended for reporting animal research, and Sex and Gender in Research

Laboratory animals	BABL/c athymic nude mice (female, 2-3 weeks) were obtained from Shanghai SLAC Laboratory Animal Co., Ltd. All mice were housed in a specific pathogen-free environment at 21±1°C and 60±5% humidity, with a 12h light-dark cycle.
Wild animals	Wild animals were not involved in this study.
Reporting on sex	The findings apply to both male and female animals.
Field-collected samples	Field-collected samples were not involved in this study.
Ethics oversight	All animal use and studies were performed according to the relevant ethical regulations. The procedures conducted on animals were approved by the Institutional Animal Care and Use Committee (IACUC) of Zhejiang University (IACUC number: IACUC-s19-026, project number: 19NGYX087Nu). The maximal tumour size/burden permitted by the IACUC is 20 mm in diameter; no mice met this criterion. Following IACUC guidelines from Zhejiang University, weight loss of more than 20%, body conditioning score (BCS) of 2 or less, or mice exhibiting signs of hunched posture, impaired locomotion or respiratory distress are criteria followed for prompt euthanasia.

Note that full information on the approval of the study protocol must also be provided in the manuscript.

Flow Cytometry

Plots

Confirm that:

☒ The axis labels state the marker and fluorochrome used (e.g. CD4-FITC).

☒ The axis scales are clearly visible. Include numbers along axes only for bottom left plot of group (a 'group' is an analysis of identical markers).

☒ All plots are contour plots with outliers or pseudocolor plots.

☒ A numerical value for number of cells or percentage (with statistics) is provided.

Methodology

Sample preparation	Flow cytometry analysis of cell apoptosis: Huh7 cells were cultured in 6-well plates (200000 cells/per well) for 24 h. Afterwards, the culture mediums were respectively treated with NMPNs, PNPs and cisplatin (20 μg/mL), and incubated at pH 6.5 for another 24 h. Cells were collected and incubated with a FITC Annexin V Apoptosis Detection Kit (BD PharmingenTM, USA) for 15 min at room temperature, an then resuspended with 400 μL 1×binding buffer. The intensity of fluorescence was measured by flow cytometer (CytoFLEX LX, USA) within 1 h.
Instrument	CytoFLEX LX
Software	CytoFLEX LX
Cell population abundance	By stained with specific fluoresence-labeled antibodies, cells were separated into different parts and cytometry can calculate the normal or apoptosis fractions because of their damaged conditions.
Gating strategy	For cell apoptosis detection, Annexin V-FITC was used to label apoptotic and necrotic cells, and PI was used to label necrotic cells. PBS-treated cells were stained with/without Annexin-V-FITC or PI to determine gate. For intracellular ROS level detection, 1×PBS-treated cells were stained without DCFH-DA to determine an appropriate gate.

☒ Tick this box to confirm that a figure exemplifying the gating strategy is provided in the Supplementary Information.
